# Cortical Neural Synchronization Underlies Primary Visual Consciousness of Qualia: Evidence from Event-Related Potentials

**DOI:** 10.3389/fnhum.2016.00310

**Published:** 2016-06-30

**Authors:** Claudio Babiloni, Nicola Marzano, Andrea Soricelli, Susanna Cordone, José Carlos Millán-Calenti, Claudio Del Percio, Ana Buján

**Affiliations:** ^1^Department of Physiology and Pharmacology “Vittorio Erspamer”, Sapienza University of RomeRome, Italy; ^2^Department of Neuroscience, IRCCS San Raffaele PisanaRome, Italy; ^3^Department of Integrated Imaging, IRCCS SDNNaples, Italy; ^4^Department of Motor Sciences and Healthiness, University of Naples ParthenopeNaples, Italy; ^5^Gerontology Research Group, Department of Medicine, Faculty of Health Sciences, University of A CoruñaA Coruña, Spain

**Keywords:** primary visual consciousness, qualia, high-resolution electroencephalography (EEG), event-related potentials (ERPs), cortical neural synchronization, dorsal visual stream, ventral visual stream

## Abstract

This article reviews three experiments on event-related potentials (ERPs) testing the hypothesis that primary visual consciousness (stimulus self-report) is related to enhanced cortical neural synchronization as a function of stimulus features. ERP peak latency and sources were compared between “seen” trials and “not seen” trials, respectively related and unrelated to the primary visual consciousness. Three salient features of visual stimuli were considered (visuospatial, emotional face expression, and written words). Results showed the typical visual ERP components in both “seen” and “not seen” trials. There was no statistical difference in the ERP peak latencies between the “seen” and “not seen” trials, suggesting a similar timing of the cortical neural synchronization regardless the primary visual consciousness. In contrast, ERP sources showed differences between “seen” and “not seen” trials. For the visuospatial stimuli, the primary consciousness was related to higher activity in dorsal occipital and parietal sources at about 400 ms post-stimulus. For the emotional face expressions, there was greater activity in parietal and frontal sources at about 180 ms post-stimulus. For the written letters, there was higher activity in occipital, parietal and temporal sources at about 230 ms post-stimulus. These results hint that primary visual consciousness is associated with an enhanced cortical neural synchronization having entirely different spatiotemporal characteristics as a function of the features of the visual stimuli and possibly, the relative qualia (i.e., visuospatial, face expression, and words). In this framework, the dorsal visual stream may be synchronized in association with the primary consciousness of visuospatial and emotional face contents. Analogously, both dorsal and ventral visual streams may be synchronized in association with the primary consciousness of linguistic contents. In this line of reasoning, the ensemble of the cortical neural networks underpinning the single visual features would constitute a sort of multi-dimensional palette of colors, shapes, regions of the visual field, movements, emotional face expressions, and words. The synchronization of one or more of these cortical neural networks, each with its peculiar timing, would produce the primary consciousness of one or more of the visual features of the scene.

## Primary Visual Consciousness: Where in the Brain?

Edelman (2003) coined the term primary consciousness referring to the ability of humans and other animals to integrate, time-by-time, perceptual and motor events together with the semantic memory for understanding the main features of the actual scene. This term is useful to distinguish perceptual awareness from higher-order consciousness, the latter being the ability to integrate the current scene with own personal history, own beliefs, etc.

Primary consciousness regards all common sensory channels such as vision, hearing, somatotopy, olfaction, taste, and equilibrium. However, the primary consciousness for the visual stimuli (objects, faces, locations, etc.) has been particularly attractive for cognitive neuroscientists, maybe because the general anatomical and functional properties of the visual system are quite known (Tononi and Koch, 2008), as a useful starting point for the challenge. Furthermore, the visual system is one of the most important perceptual channels for exploring the environment in humans. Overall, “primary visual consciousness” can be defined as a process underlying an immediate self-report about the core features of an adequate visual stimulation. According to Pinker (1997), that self-report implies subjective phenomenal experience and the ability to describe it.

What are the neural correlates of the primary visual consciousness? The main issue is “where” there are neurons in the brain whose activity is related to primary visual consciousness. Crick and Koch (2003) defined neural correlates of the primary visual consciousness as “the minimal set of neuronal events that give rise to a specific aspect of a conscious percept”. In their view, the primary visual consciousness depends on several coalitions or nodes of neurons that rest on the properties of highly elaborate neural networks. The smallest nodes in these neural networks might be cortical microcolumns (or minicolumn) and macrocolumns (or columns, hypercolumns, modules) in the visual cerebral cortex. More specifically, the cortical microcolumns are formed by a group of 50–100 neurons having nearly identical receptive fields and encoding very similar features of the stimulus (Buxhoeveden and Casanova, 2002). About 50–100 cortical microcolumns form a macrocolumn in the cerebral cortex (Horton and Adams, 2005). Interestingly, the neurons of a macrocolumn have nearly identical receptive fields and can be penetrated orderly by a microelectrode inserted orthogonally to the surface of the cerebral cortex. These neurons encode a full set of features of the stimulus possibly associated with the mental representation of the stimulus characteristics (“qualia”) during primary visual consciousness. Keeping in mind the above considerations and data, it can be speculated that primary visual consciousness would emerge with the contribution of brain neural correlates at the mesoscale spatial level, corresponding to local neural populations in the cortical macrocolumns and microcolumns (Crick and Koch, 2003).

Apart from the mesoscale level, several neuroscientists have proposed theories on the neural correlates of primary visual consciousness implying the contribution of cerebral neural correlates at macroscale spatial level, corresponding to gross neural pathways in the brain. The so-called “globalists” posited that primary visual consciousness emerges from the inter-related activation of vast areas of the brain. In contrast, the so-called “localizationists” proposed that visual consciousness does emerge from specific and relative small brain circuits (see Kouider, 2009). Examples of the globalist theories are the Dynamic Core theory by Edelman and Tononi (2000) and the Global Neuronal Workspace theory by Dehaene and Naccache (2001) and Dehaene et al. (2003). These theories propose that primary visual consciousness is the byproduct of a global, widespread neural activity in thalamocortical circuits in the brain. This widespread brain neural activity would explain the internal feeling of “unicity” and “globality” of the experience of primary visual consciousness. On the other hand, examples of the localizationist theories are those by Zeki and Bartels (1999), Milner and Goodale (2006), and Lamme (2010). Zeki and Bartels (1999) and Zeki (2003) proposed the Microconsciousnesses theory, positing that the conscious perceptual representation of visual objects is formed by many visual “microconsciousnesses” emerging from the activity distributed in space and time into several specialized visual areas. In the same vein, Milner and Goodale (2006) presented the Duplex Vision theory, positing that the conscious perceptual representation of visual objects emerges from the activity of the ventral visual pathway in the cerebral cortex while the consciousness of the visuospatial object features is subserved by the cortical dorsal visual pathway. As a development of this localizationist view, Lamme (2010) published the Local Recurrence Theory, positing that primary visual consciousness emerges from the local recurrent exchange of signals between higher- and lower-level visual areas in the cortical ventral stream.

The distributed brain neural populations involved in the primary visual consciousness raise the issue of “how” the activities of these areas are bound and coordinated with each other, namely the so-called binding problem.

## The Binding Problem of the Primary Visual Consciousness

It can be speculated that the solutions of the binding problem for the primary visual consciousness have to occur not only at the level of cortical macrocolumns and microcolumns (mesoscale), but also at the levels of synapsis (microscale) and gross cerebral circuits (macroscale). The binding of the neurons at different spatial scales might be facilitated or disrupted by neural signals (i.e., action potentials and release of neurotransmitters) synchronizing in time the activity of these neurons (i.e., action potentials and post-synaptic potentials) in cycles of excitation and inhibition. These cycles denote the emerging feature of the brain, namely its oscillatory nature.

An example of the neurophysiological oscillatory mechanism operating at different spatial scales (i.e., synapsis, neural populations, and brain regions) is the generation of the gamma rhythm in the visual cortex during feature analysis of visual stimuli. This gamma rhythm is characterized by oscillations in the cortical local field potentials at about 40 Hz, with alternating positive and negative voltage peaks (macroscale). According to Engel et al. (1999) and Engel and Singer (2001), these positive and negative voltage peaks reflect the temporal synchronization of action and post-synaptic potentials at about 40 Hz in cortical and thalamocortical neurons located in lateral geniculate nucleus (LGN), namely the relay-mode neurons whose receptive fields are nearly overlapping and simultaneously stimulated by an adequate visual stimulus feature (mesoscale). The synchronous generation of action and post-synaptic potentials at about 40 Hz in those neurons does establish a neural network (neural coalition) and is thought to be related to primary visual consciousness of that stimulus feature (see for example binocular rivalry process; Engel et al., 1999; Engel and Singer, 2001).

Another example of the neurophysiological oscillatory mechanism operating at different spatial scales is the generation of the classical alpha rhythm recorded in visual cortex during quiet wakefulness (macroscale). This alpha rhythm is characterized by large oscillations at about 10 Hz, with alternating positive and negative voltage peaks. According to Hughes et al. (2004, 2008, 2011) and Lörincz et al. (2008, 2009), these voltage peaks are generated by cortical pyramidal neurons due to synchronizing signals released by 70% of the relay-mode neurons firing in the tonic mode (mesoscale). These thalamocortical neurons are inhibited by GABAergic LGN inter-neurons (mesoscale). In their turn, the GABAergic inter-neurons are excited by 30% of thalamocortical LGN neurons, namely the high threshold neurons firing in the bursting mode (mesoscale). The population of the high threshold thalamocortical LGN neurons shows a collective oscillating behavior, due to a fast propagation of action potentials by electric synapses served by gap junction proteins (microscale). Those high threshold thalamocortical LGN neurons would be excited by glutamatergic and cholinergic neurotransmitters released by cortical and sub-cortical neurons (Hughes et al., 2008; Lörincz et al., 2008). It can be speculated that this neurophysiological oscillatory mechanism regulates arousal and selective attention processes in the visual cortex, and may play some role in primary visual consciousness (Babiloni et al., 2014).

An interesting feature of the neurophysiological oscillatory mechanisms is that more than one rhythm may co-occur in the same neural population. An enlightening example is the coding of spatiotemporal trajectories during exploratory movements in rodent hippocampus (Zheng et al., 2016). In rats, hippocampal place neurons (mesoscale) emitted action potentials co-occurring at theta (4–8 Hz) and low-frequency gamma (around 40 Hz) rhythms to retrieve neural representations of sequences of locations to reach sometime after. Differently, they emitted action potentials co-occurring at theta and high-frequency gamma (around 80 Hz) rhythms immediately before the actual trajectory to execute in that place. It was proposed that theta/gamma oscillations sub-serve the encoding of multiple items of information in a serial order not only in the hippocampus for spatial contents but also in other cerebral regions engaged in sensory integration and working memory (Lisman, 2005; Babiloni et al., 2009a). In that theoretical framework, theta rhythm might provide an absolute phase reference to preserving the encoding order while some gamma cycles into a theta cycle would encode the single items to be memorized (Lisman, 2005).

Keeping in mind the above data and considerations, a reasonable theory is that multiple neurophysiological oscillatory mechanisms synchronize brain neurons at different spatial scales to regulate brain arousal, activate a selective enhancement of the stimulus feature extraction, and produce the mental experience called primary visual consciousness. However, the methodological approaches by the reported findings have some limitations in the spatial and temporal resolution of the neural correlates of the primary visual consciousness in humans. Concerning the spatial resolution, limited regions of the brain were explored simultaneously in the mentioned animal studies (i.e., LGN, hippocampus, entorhinal cortex, visual cortex), so the neurophysiological binding of remote and distributed brain networks could not be investigated. Furthermore, just one cycle of the theta and alpha rhythms takes about 200 and 100 ms, respectively. Therefore, it was not possible to study the temporal evolution of the cortical neural synchronization millisecond-by-millisecond in relation to the mental experience of primary visual consciousness. To fill this gap, high-resolution electroencephalography (EEG) enables the study of cortical neural synchronization related to primary visual consciousness with those ideal characteristics.

## High-Resolution Electroencephalography (EEG)

Recording of EEG activity from scalp electrodes is typically used to unveil temporal and spatial information about cortical neural synchronization and de-synchronization mechanisms underlying quite wakefulness or the processing of sensory, cognitive, and motor events (Nuwer, 1988; Steriade, 2006). During and immediately after these events, changes in the spontaneous EEG activity can be observed. Averaging the EEG activity accompanying the single events repeated tens and tens of time produces a typical sequence of positive and negative voltage peaks, the so-called event-related potentials (ERPs; Luck, 2005; Murray et al., 2008).

Spontaneous EEG rhythms may reflect synaptic neural currents associated with excitatory and inhibitory post-synaptic potentials in large populations of cortical pyramidal neurons whose summation produces the local field potentials recordable by intra-cortical electrodes (Nunez, 2000; Michel et al., 2004; Rossini et al., 2007; Babiloni et al., 2009a). These EEG rhythms mainly result from the spatial and temporal summation of extracellular local field potentials generated in superficial cortical layers. In the superficial cortical layers, synaptic neural currents would reflect the synaptic activity of pyramidal neurons located in the cortical gyri, whose dendrites are oriented radially to the scalp surface (Nunez and Srinivasan, 2006). Whereas, synaptic neural currents of the pyramidal neurons located in the cortical sulci would propagate tangentially to the scalp surface, providing a minor contribution to the generation of on-going scalp EEG rhythms (Nunez, 2000). Spontaneous EEG activity is usually recorded to assess states of vigilance or consciousness like sleep and resting state wakefulness at eyes closed or open. The analysis of spontaneous EEG rhythms can reveal resting state oscillatory activity, epileptic seizures or sleep spindles. Such analysis is performed in the frequency domain, and typically implies the computation of EEG power density at the electrode and/or the quantification of the functional connectivity of EEG rhythms recorded from different electrodes.

The ERPs may reflect coordinated neural network activity and can be considered as a series of transient post-synaptic responses of main pyramidal neurons triggered by a specific stimulus (Pfurtscheller and Lopes da Silva, 1999). The physiological basis of cortical ERPs arises from synchronous interactions among large numbers of participating neurons, including local interactions involving excitatory pyramidal neurons and inhibitory interneurons, as well as long-range interactions mediated by axonal pathways in the white matter (Lopes da Silva, 1991; Bressler, 2002). Measurement of ERPs requires averaging of many EEG epochs that are phase- and time-locked to sensory, cognitive or motor “events,” in order to remove nonphase-locked EEG rhythms from ERPs. Event-related cortical activity can be quantified by measuring latencies and amplitudes of distinct ERP components in the time domain.

On the other hand, the spatial resolution of EEG techniques depends on the amount of EEG channels, and also on mathematical procedures to model cortical sources of EEG activity and to take into account the properties of the head as a volume conductor (Nunez, 2000). High-density EEG using between 48 and 256 electrodes, along with proper mathematical procedures, can achieve a spatial resolution in the order of few centimeters. Regularized linear procedures are typically used for solving the inverse problem, which provides an approximation of current density at the cortical source models on the basis of the head volume conductor properties and of the voltages/current density at the scalp locations (Pascual-Marqui and Michel, 1994; Grech et al., 2008; Rossetti and Kaplan, 2010). Cortical sources of the resting state EEG rhythms or ERPs can be reliably estimated by low-resolution brain electromagnetic tomography analysis (LORETA^1^; Pascual-Marqui and Michel, 1994; Pascual-Marqui et al., 1999, 2002). LORETA is a functional imaging technique estimating smoothed linear inverse solutions accounting for distributed EEG sources within Talairach space (Pascual-Marqui et al., 2002). This software computes 3-D linear solution for the EEG inverse problem within a three-shell spherical head model including the scalp, skull, and brain compartments. The brain compartments are restricted to the cortical gray matter/parahippocampal regions and are co-registered to the Talairach probability brain atlas. This compartment includes 2394 voxels (7 mm resolution), each voxel containing an equivalent current dipole. A better solution for source localization can be achieved by eLORETA and sLORETA than original LORETA (Pascual-Marqui et al., 2002; Pascual-Marqui, 2007). eLORETA uses a realistic head model (Fuchs et al., 2002) using the MNI152 template (Mazziotta et al., 2001), with a three-dimensional solution space restricted to cortical gray matter and electrode coordinates provided by Jurcak et al. (2007). The intracerebral volume is partitioned in 6239 voxels with 5 mm spatial resolution. Thus, eLORETA images obtained from EEG data represent the electrical activity at each voxel in the neuroanatomic Montreal Neurological Institute (MNI) space as the exact magnitude of the estimate current density. Anatomical labels as Brodmann areas are also reported using MNI space, with adaptation to Talairach space (Brett et al., 2002).

## A Scientific Program to Investigate ERP Cortical Sources During Primary Visual Consciousness in Humans

In the past years, we have developed a scientific program using high-resolution EEG to investigate ERP cortical sources related to primary visual consciousness in healthy adults. The main hypothesis was that ERP source activity would reflect the fine (milliseconds) time evolution in the cortical neural synchronization related to primary visual consciousness. Furthermore, it was expected that the ERP source activity would unveil the role of this synchronization in different brain regions as a function of the diverse “qualia” characterizing the conscious experience (Babiloni et al., 2014).

In the scientific program, the same basic stimulus paradigm was used to investigate the primary visual consciousness for three basic visual features such as visuospatial, face emotions, and written words. The stimulus paradigm was based on the following sequence of visual stimuli: (i) the background masking stimulus (forward masking hiding the cue); (ii) the cue stimulus (the stimulus to self-report at the end of the trial); (iii) the background masking stimulus (backward masking hiding the cue); and (iv) the target stimulus (the “go” stimulus eliciting a hand motor response, namely pressing a mouse button).

In all the quoted experiments, the duration of the cue stimuli was determined subject-by-subject with a preliminary short behavioral test lasting few minutes (no EEG recording). In that test, the subject received the cue stimuli with different duration on a computer monitor and had to provide a hand response on a computer button. The subjects used the hand response to communicating if the cue stimulus had been seen. The sequence of the cue stimuli was planned to mix the cue stimuli with different duration in a pseudorandom order. This procedure avoided learning effects during the session.

A dedicated software computed the percentages of “seen” cue stimuli for every stimulus durations. Based on these data, the experimenters selected the cue stimulus duration nearest to 50% of “seen” cases in that particular subject (threshold time). On the other hand, the “optimal” duration of the cue stimulus was always kept fixed on a given subject during the second part of the experiment, namely the phase of the EEG recording.

Threshold time of the cue stimuli was slightly different in our three experiments. Specifically, the mean (standard error mean, SE) threshold time of the cue stimuli was 101 ms (8.8) in the “visuospatial” experiment, 64.6 ms (9.1) in the “face expression” experiment, and 37.2 ms (2.7) in the “written words” experiment. In the “visuospatial” experiment, we performed a control analysis showing that the percentage of “seen” trials did not change during three different periods of the EEG experiment (initial, intermediate, and final). This control finding suggested that in the present experimental conditions, learning and fatigue processes may not play a major role in the ability of the brain to produce primary consciousness in about 50% of the (cue) visual stimuli. This finding validated the choice of the “optimal” stimulus duration in the preliminary phase, before the phase of EEG recordings.

In the “visuospatial” experiment, the cue stimulus feature of interest was the localization of a circle at the right or left side of a computer screen. The “go” stimulus (a circle at left or right side of the computer) elicited the click on the right or left mouse button. In the “face expression” experiment, the cue stimulus feature of interest was the expression of the mouth in an emoticon (happy, sad or neutral). The “go” stimulus (an emoticon with the mouth conveying happy or sad expression) elicited the click on the right or left mouse button as a function of the emotional expression represented in the emoticon. In the “written word” experiment, the cue stimulus feature of interest was one of four words. The “go” stimulus (the same words) elicited the click on the right or left mouse button as a function of the semantic meaning of the words (i.e., living being vs. not living being). Immediately after the hand motor response, the subjects had to say “seen” if they had detected the cue stimulus (“seen” trial) or “not seen” if they had missed it (“not seen” trial”).

In all our experiments on primary consciousness, the instructions for subjects emphasized to balance accuracy and speed in the hand motor response immediately after the “go” stimuli. Of note, the subjects communicated “seen” or “not seen” for the cue stimulus verbally only after their hand response to the “go” (target) stimulus. Therefore, they had some seconds to prepare the verbal response “seen” or “not seen” in the period from the appearance of the cue stimulus to the hand motor performance following the “go” stimulus. This design minimized the effects of uncertainty, confusion, and impulse responses in the communication of the verbal response “seen” or “not seen”. Of note, alternative procedures can be better when the degree of awareness is of interest for the working hypothesis. In this case, it is recommended the use of a fine-grained scale to recollect the subject’s impression about the visual stimulus. For example, a reference experiment on primary visual consciousness used a 4-point subjective scale (Perceptual Awareness Scale, PAS) including the following levels: “no experience of the stimulus”, “brief glimpse of the stimulus but could not recognize what it was”, “almost clear impression of the stimulus”, and “clear impression of the stimulus” (Melloni et al., 2011).

The basic concept of the paradigm in the above three experiments is that a statistical difference in the ERP source activity between “seen trials” and “not seen trials” would confirm the hypothesis that primary visual consciousness is strictly associated with an enhanced cortical neural synchronization. In the case of a difference in the ERP peak latency, such difference would unveil a specific timing of the neural correlates of the primary visual consciousness. In the case of a difference in the ERP source topography, such difference would unveil the cortical regions associated with primary visual consciousness of the given stimulus feature.

**Figure 1:**
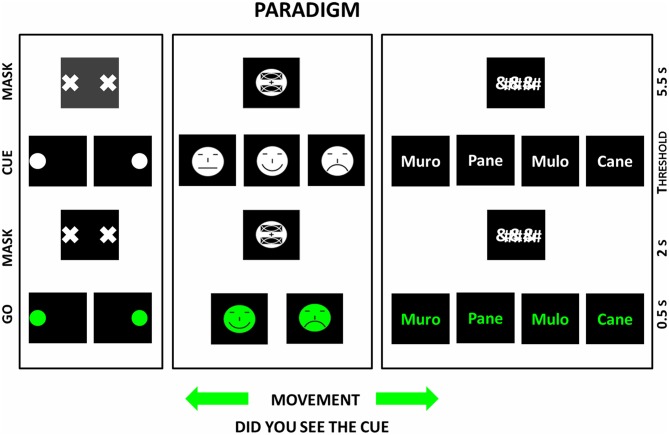
**Sequence of visual stimulus events during the paradigm used in three studies on primary visual consciousness whose results are reviewed in the present article (Babiloni et al., 2006, 2010, under major revision in 2016).** In those studies, the sequences of visual stimuli are represented in the left (“visuospatial” experiment), middle (“face expression” experiment), and right (“written words” experiment) parts of figure. In the vertical axis of the diagrams, the sequence of the visual stimuli is illustrated. In the three studies, the same basic stimulus paradigm was used. The stimulus paradigm was based on the following sequence of visual stimuli: the cue (the stimulus to self-report at the end of the trial), the mask (hiding the cue), and the target (the “go” stimulus eliciting a hand motor response, namely pressing a mouse button). Importantly, the duration of the cue stimulus was defined individual-by-individual with a preliminary short experiment. The duration of the cue stimulus had to induce a primary visual consciousness (i.e., self-report) of the stimulus feature of interest in about 50% of cases. In the “visuospatial” experiment, the cue stimulus feature of interest was the localization of a circle at the right or left side of a computer screen. The “go” stimulus (a circle at left or right side of the computer) elicited the click on the right or left mouse button. In the “face expression” experiment, the cue stimulus feature of interest was the expression of the mouth in an emoticon. The “go” stimulus (a mouth conveying happy or sad face expression) elicited the click on the right or left mouse button. In the “written words” experiment, the cue stimulus feature of interest was one of some words. The “go” stimulus (the same words) elicited the click on the right or left mouse button as a function of the semantic meaning of the words (i.e., living being vs. not living being). Immediately after the hand motor response, the subjects had to say “seen” if they had detected the cue stimulus (“seen trial”) or “not seen” if they had missed it (“not seen trial”).

Figure 1 illustrates the basic sequence of the cue, mask, and go stimuli in the above three experiments. The main results of those experiments are shortly reported and discussed in the following sections. More details can be found in the original articles (Babiloni et al., 2006, 2010, under major revision in 2016).

## The Experiment on the ERPs Accompanying the Primary Consciousness of Visuospatial Stimuli

**Figure 2:**
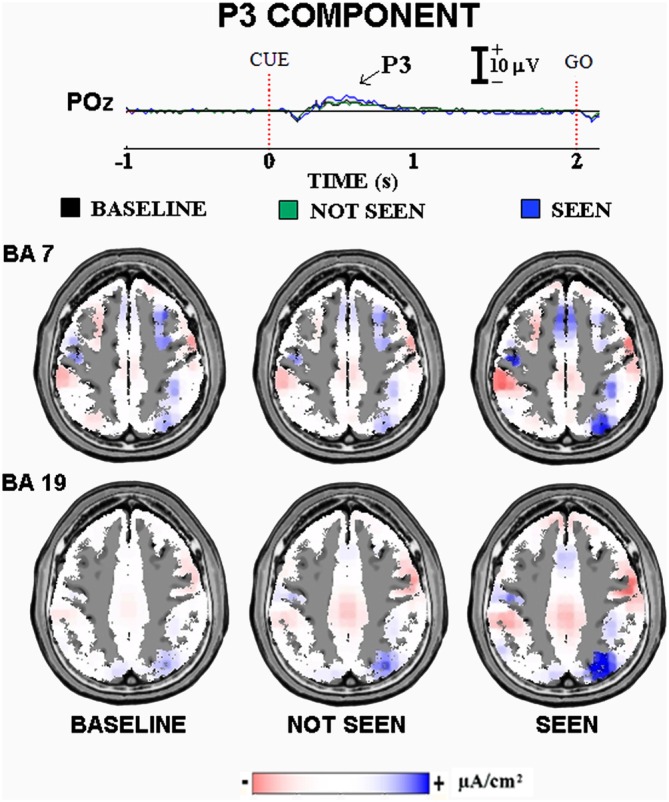
**Core results of the experimental paradigm used in the study of Babiloni et al. (2006).** Top: waveforms of the grand averaged (*N* = 12) event-related potentials (ERPs) are shown at POz electrode for the three classes of events of this experiment on visuospatial primary visuospatial consciousness, namely “baseline” (cue stimulus not presented after the central fixation stimulus as a control condition), “not seen” (cue stimulus not detected), and “seen” (cue stimulus detected) trials. Bottom: the grand averaged low-resolution brain electromagnetic tomography (LORETA) solutions modeling the distributed source activity for P3 component of the ERPs are shown. These solutions were based on the electroencephalographic (EEG) voltages recorded from all 128 recording electrodes at the P3 peak latency. They concerned the three classes of the mentioned events separately. For more details, see the main text and the original article (Babiloni et al., 2006).

Figure 2 illustrates the main results of our “visuospatial” experiment on the ERPs accompanying primary visual consciousness. As mentioned above, the cue stimulus feature of interest was the localization of a circle at the right or left side of a computer screen. The post-stimulus ERP waveforms showed the highest amplitude at the parieto-occipital midline. In Figure 2, the ERP waveforms refer to the POz electrode (10–20 system) and unveil the standard sequence of positive (P) and negative (N) voltage peaks typically following simple visual stimuli. Specifically, three main ERP components were found, namely the N1, P2, and P3. The peaks of these components were well visible in the ERPs formed averaging separately the three classes of trials occurring in the experiment, namely the “seen”, the “not seen”, and the “baseline” trials. The “baseline” trials had a control purpose. In these trials, after the background masking stimulus, a black screen instead of a circle was presented. The “baseline” trials allowed controlling the reliability of the subjects’ self-report (i.e., the percentage of the self-report “not seen” in the “baseline” trials) and the ERPs (i.e., similar ERPs were expected in the “not seen” and in the “baseline” trials). Indeed, the control results confirmed the good reliability of the subjects’ self-reports and the ERPs in the present experimental conditions. The presence of ERPs in the “baseline” trials should not be a surprise, since these ERPs were indeed evoked by the change in the visual scene from the background masking stimulus to the black screen.

An interesting result of the “visuospatial” experiment was the lack of statistical difference (*p* > 0.05) in the latency of the N1, P2, and P3 peaks when the ERPs accompanying the “seen” trials were compared with those accompanying the “not seen” trials. This result points to the same timing and stages of cortical neural synchronization in the “seen” and “not seen” trials, regardless the primary visual consciousness. However, the neural correlates of the primary visual consciousness were not useless. The reaction time of the “go stimuli” was faster in the “seen” than the “not seen” trials as whether the primary consciousness is associated with an enhanced information processing of visuospatial stimuli. Noteworthy, this difference was not due to the lack of any beneficial effect of the cue stimuli in the “not seen” trials. Indeed, the cue stimuli speeded the reaction time of the “go stimuli” even in the “not seen” trials, pointing to an ability of the brain to extract and use spatial information from the cue stimuli not only in association with primary consciousness but also outside of it.

When did the cortical neural synchronization show some difference in relation to the primary consciousness of visuospatial stimuli? In the “visuospatial” experiment, the parietal P3 component peaking about at 400 ms post-stimulus was higher in amplitude in the “seen” than the “not seen” trials (*p* < 0.05). This result suggests that human brain does process simple visuospatial stimuli with enhanced cortical neural synchronization around 400 ms post-stimulus in association with the primary consciousness. Interestingly, this result cannot be explained by the physical features of the cue stimuli, as the “seen” and “not seen” trials were physically identical (i.e., duration, color, shape, size, brightness, contrast, etc.). Furthermore, there was no effect of the motor response, as the cue stimulus required no immediate behavioral response. Indeed, the subjects had to produce a simple motor response after the “go” stimulus in both “seen” and “not seen” trials.

Where did the cortical neural synchronization show some differences in relation to the primary consciousness of visuospatial stimuli? The source analysis (LORETA) of the parietal P3 component pointed to higher current density in extrastriate occipital and posterior parietal areas in the “seen” than the “not seen” trials (*p* < 0.05, Figure 2). This result suggests that human brain processes simple visuospatial stimuli with an enhanced cortical neural synchronization in extrastriate occipital and posterior parietal areas around 400 ms post-stimulus in association with the primary consciousness.

Keeping in mind the above findings, the “visuospatial” experiment disclosed a quite similar temporal evolution of the cortical neural synchronization in the occipital and parietal cortical “dorsal” stream for the processing of visuospatial stimuli with and without primary consciousness. However, the primary consciousness of those stimuli was associated with an enhanced neural synchronization in that cortical pathway around 400 ms post-stimulus. Furthermore, the behavioral performance was better in the trials associated with the primary consciousness when compared to those in which the cue stimuli were not perceived consciously.

The results of our “visuospatial” experiment extended previous evidence on ERPs and primary visuospatial consciousness. The extension concerned the estimation of ERP cortical sources and the fine control of the motor aspects of the task (Babiloni et al., 2006). The findings of this experiment specified the topography in parietal and occipital cortex of the effects of primary visuospatial consciousness without motor confounds (Babiloni et al., 2006). Other studies documented differences between “seen” and “not seen” trials in ERPs distributed over widespread posterior scalp regions at 200–400 ms after the presentation of different types of stimuli (visuospatial, letters, gabor patchs, etc.) with paradigms of binocular rivalry, attentional blink, visual masking, threshold detection tasks, etc. (see Railo et al., 2011 for a review). All those ERP studies showed an increased P3 amplitude in widespread scalp regions in relation to primary visual consciousness (Fernandez-Duque et al., 2003; Del Cul et al., 2007; Lamy et al., 2009; Chica et al., 2010), especially when experimental demands did not induce a stimulus expectancy (Melloni et al., 2011). In this context, the topographical specificity of our findings was confirmed by magnetoencephalographic (MEG) evidence (Liu et al., 2012). That MEG study reported cortical source activity in bilateral occipital-temporal (but not parietal) regions at about 200–350 ms post-stimulus in association with primary visual consciousness of non-spatial stimuli (Liu et al., 2012). The findings of our “visuospatial” experiment corroborated the involvement of parietal and occipital cortex in the primary visuospatial consciousness (Babiloni et al., 2006), thus complementing the previous view on the functional role of parietal cortical areas in visuo-spatial attention and consciousness (Driver et al., 2001; Chica et al., 2010).

It should be remarked that in our “visuospatial” experiment, primary consciousness concerned the appearance or not (subjective awareness) of the visual stimulus with no requirement to specify its characterizing features for control purposes. This methodology reduced anxiety and uncertainty in the response but did not permit a strict direct control of its validity. However, an indirect control was provided by converging evidence. There was a low rate of false positive recognitions (around 3%) and the beneficial covert cue effects on the mean reaction times to the “go” stimuli (faster reaction time for the “go” stimuli appearing from the same side of the “seen” cue stimuli). Another confirmation of the reliability of the self-report in these threshold experiments was indirectly given by Lamy et al. (2009). They reported that a widespread posterior P3 was higher in amplitude for “seen” stimuli associated with the primary visual consciousness and correct localization when compared to “not seen” stimuli. Overall, these findings confirmed the general reliability of the self-report and the objective measures in our experiment.

The ERP results of our “visuospatial” experiment suggested that primary visual consciousness of spatial qualia is mainly related to a late stage of the brain stimulus information processing, namely at the time of the P3 component (i.e., 200–400 ms post-stimulus). This finding is in line with several ERP studies mentioned above (see Railo et al., 2011 for a review). However, other ERP studies found differences between “seen” and “not seen” visual stimuli in posterior cortical areas near 100 ms post-stimulus, at around the peak of P1 component (Pins and Ffytche, 2003; Mathewson et al., 2009). Furthermore, MEG techniques with a high spatial resolution reported event-related magnetic fields accompanying primary visual consciousness in contralateral temporal cortex at around 120 ms post-stimulus, showing a dissociation from spatial attention processes (Wyart et al., 2012). Despite this very interesting previous evidence, more research is needed to clarify spatial, temporal, frequency, and phase features of neurophysiological mechanisms and brain neural circuits underpinning the complex interaction between consciousness and other factors such as visual stimulus expectancy and physical visibility, spatial pre-attentive and attention processes, and paradigm time constraints and cognitive load (Lavie, 2006; Wyart and Tallon-Baudry, 2008; Melloni et al., 2011; Liu et al., 2012; Wyart et al., 2012).

In this vein, another “visuospatial” experiment of our group required subjects to provide a hand response when a central “go” (target) appeared (Babiloni et al., 2009b). The subjects had to press the button located in the side opposite (mental inversion of the spatial location) to that of a visual cue delivered at threshold time to produce “seen” and “not seen” trials. Instructions asked a hand response in one side in any case, even when the cue was not consciously perceived. Results showed that ERP sources were higher in activity for the “seen” than “not seen” trials from 100–400 ms post-stimulus. Source topography of this ERP activity was located not only in occipital and parietal cortical areas but also in the prefrontal cortex, particularly in the latency of the P3 component (Babiloni et al., 2009b). More research is needed to disentangle the early neural correlates of primary visual consciousness from preconscious modulation of stimulus features, depending on the probability of a subsequent self-report (Pins and Ffytche, 2003; Koivisto et al., 2008).

In addition to the studies on healthy subjects, the results of our “visuospatial” ERP experiment also complement and maybe enlighten neurophysiological underpinning of previous studies on abnormal primary consciousness of visuospatial stimuli in neurological subjects with visuospatial neglect and visual extinction, namely deficits in the spatial awareness for stimuli on the side opposite to a brain lesion (Driver et al., 1997; Vuilleumier et al., 2001). Visual extinction is often present in patients with focal parietal lesions, typically in the right posterior parietal cortex, whereas the primary sensory pathways are intact. It is characterized by a spared ability to detect a stimulus presented unilaterally on either hemifield, but if the stimulation is bilateral, the stimulus presented in the ipsilateral field is detected while that on the contralateral field is “extinguished” from consciousness. Both behavioral and neuroimaging results have found that extinguished visual stimuli may be processed in patients with extinction. The reaction times to target stimuli on the ipsilesional visual hemifield is affected by concurrent undetected stimuli on the contralesional visual hemifield (Marzi et al., 1996; Vuilleumier and Rafal, 2000). Moreover, visual extinction is affected by the semantic relationship between concurrent ipsilesional (“seen”) and contralesional (“extinguished” or “not seen”) stimuli (Baylis et al., 1993; Mattingley et al., 1997; Vuilleumier and Rafal, 2000).

Further relevant data come from previous functional magnetic resonance imaging (fMRI) showing that the extinguished stimuli are processed in neurological patients by the same occipital and parietal areas of the “dorsal stream” exhibiting enhanced P3 source activity in the “seen” trials in our “visuospatial” experiment performed in healthy subjects (Driver, 1996; Driver et al., 1997; Heilman et al., 1997; Robertson et al., 1997; Driver and Mattingley, 1998). In the bilateral stimulation trials, those neurological patients consciously perceived only the stimuli delivered at the right hemifield, but those cortical areas were activated despite the extinction of left visual stimuli (Rees et al., 2000; Vuilleumier et al., 2001). In those neurological patients, the same occipital and parietal areas of the “dorsal stream” were maximally activated in the “seen” trials, namely the trials in which they consciously perceived both left and right visual stimuli (Marzi et al., 2000; Driver et al., 2001; Vuilleumier et al., 2001).

The results of our “visuospatial” experiment also complement those of previous ERP evidence in neurological patients with “visual extinction” (Marzi et al., 2000; Driver and Vuilleumier, 2001; Vuilleumier et al., 2001). More specifically, Marzi et al. (2000) showed the presence of ERP components in both “seen” and “not seen” trials during bilateral visual stimulation in a patient with visual extinction. Nevertheless, occipital and parietal ERPs of the right (damaged) hemisphere were higher in amplitude in “seen” than in “not seen” trials in line with the results of the present “visuospatial” experiment in healthy subjects. Furthermore, Vuilleumier et al. (2001) reported the presence of occipital early ERP components during bilateral stimulation only in the “seen” trials in another patient with “visual extinction”, whereas the late ERP components including the posterior P3 were observed in both “seen” and “not seen” trials. Indeed, we observed substantial P3 source activity in both “seen” and “not seen” trials in the healthy subjects of the present “visuospatial” experiment. A very promising clinical application of the present methodological approach will be the study of ERP sources in those neurological patients during “seen” (unilateral, bilateral) and “not seen” trials.

## The Experiment on the ERPs Accompanying the Primary Consciousness of Schematic Face Expressions

**Figure 3:**
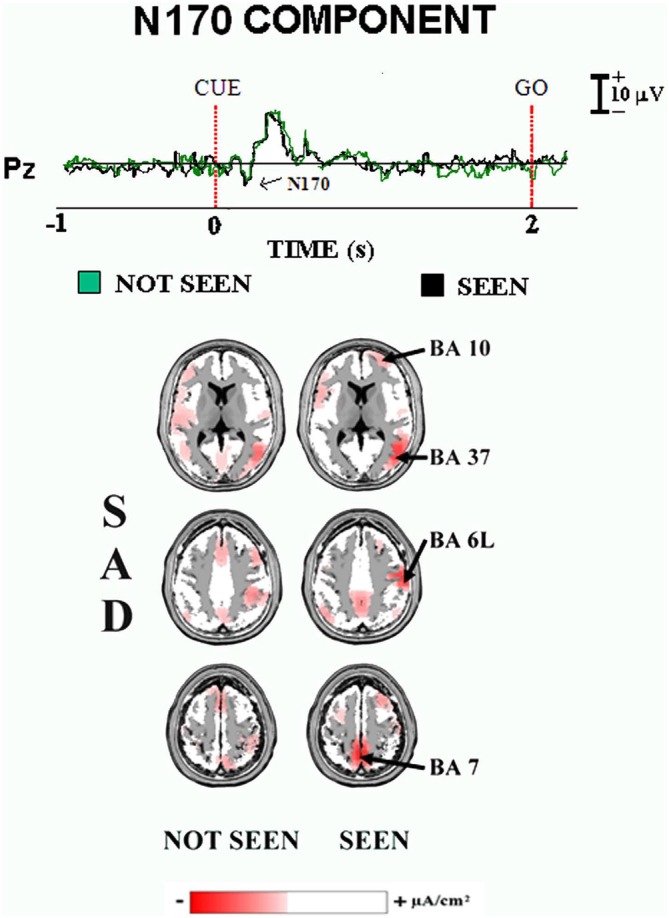
**Core results of the experimental paradigm used in the study of Babiloni et al. (2010).** Top: waveforms of the grand averaged (*N* = 15) ERPs are shown at Pz electrode for the sad condition in the “not seen” and “seen” trials. Bottom: the grand averaged LORETA solutions modeling the distributed source activity for N170 component of the ERPs are shown. These solutions were based on the EEG voltages recorded from all 128 recording electrodes at the N170 peak latency. They concerned the “not seen” and “seen” trials averaged separately. For more details, see the main text and the original article (Babiloni et al., 2010).

Figure 3 illustrates the main results of our “face expression” experiment on the ERPs accompanying primary visual consciousness (Babiloni et al., 2010). As mentioned above, the cue stimulus feature of interest was the detection of the mouth of an emoticon, representing schematic emotional face expressions (i.e., neutral, sad, and happy). The post-stimulus ERP waveforms showed a highest amplitude at the parietal midline electrodes. In the Figure 3, the group ERP waveforms refer to Pz electrode (10–20 system) and unveil the standard sequence of positive (P) and negative (N) voltage peaks typically following face stimuli. Specifically, four main ERP components were found, namely the N100, N170, P200, and P300. The peaks of these components were well visible in the ERPs formed averaging separately the two classes of trials occurring in the experiment (i.e., the “seen” and “not seen”) for the three emotional conditions (i.e., neutral, sad, and happy).

Also for the “face expression” experiment, there was a lack of statistical difference (*p* > 0.05) in the latency of the N100, N170, P200, and P300 peaks when the ERPs accompanying the “seen” trials were compared with those accompanying the “not seen” trials in a given emotional condition. This result confirmed that of the “visuospatial” experiment, and points to the same timing of cortical neural synchronization in the “seen” and “not seen” trials, regardless the primary visual consciousness and the emotions conveyed by the schematic face expressions. However, the neural correlates of the primary visual consciousness were not useless. As for the “visuospatial” experiment, the reaction time of the “go stimuli” was faster in the “seen” than the “not seen” trials, especially when the target face was “happy”, as whether the primary consciousness was associated with an enhanced information processing of visuospatial stimuli.

When did the cortical neural synchronization show some difference in relation to primary consciousness of schematic emotional face expressions? In the “face expression” experiment, the parietal N170 component peaked about at 180–200 ms post-stimulus for all three emotional face expressions (i.e., neutral, sad, and happy). Furthermore, this ERP component was higher in amplitude in the “seen” than the “not seen” trials (*p* < 0.05). This result suggests that human brain does process schematic emotional face expressions with enhanced cortical neural synchronization around 200 ms post-stimulus, possibly in association with the primary consciousness. Interestingly, N170 peak latency was earlier than that of the P3 peak in “visuospatial” experiment (e.g., 400 ms post-stimulus). This difference is suggestive of faster information processing for the primary consciousness of the schematic emotional face expressions, maybe for their obvious biological and social salience (Batty and Taylor, 2003).

Where did the cortical neural synchronization show some differences in relation to the primary consciousness of schematic emotional face expressions? As a main finding, the source analysis (LORETA) of the N170 component pointed to higher current density in prefrontal, premotor, and posterior parietal areas in the primary consciousness of the sad face expression (*p* < 0.05, Figure 3). This result suggests that human brain processes schematic emotional face expressions with an early (i.e., 200 ms post-stimulus) enhanced cortical neural synchronization in a distributed frontal and parietal network, not including temporal face processing area.

The results of the “face expression” experiment complement previous fMRI evidence showing that awareness of emotional face expressions specifically enhanced activation of bilateral parietal, temporal, and frontal areas (Vuilleumier et al., 2002). Furthermore, these results extend previous ERP evidence on N170 and N250 components associated with face stimuli (Bentin et al., 2002; Schweinberger and Burton, 2003). In the previous ERP studies, these early components were modulated by the primary consciousness of the emotional valence of both schematic and photographic facial expressions (Sokolov and Boucsein, 2000; Balconi and Lucchiari, 2005; Krombholz et al., 2007). Instead, later N400 component was modulated by the primary consciousness of the emotional valence of photographic facial stimuli (Halgren and Marinkovic, 1995; Kiefer and Spitzer, 2000). Keeping in mind the above data, the “face expression” experiment suggests that the primary consciousness of schematic face expressions may emerge from early (i.e., 200 ms post-stimulus) in relation to the activation in associative parietal and frontal cortical areas not specialized for basic face information processing and largely different from those activated by visuospatial stimuli. Again, the primary consciousness may be related to an enhanced cortical neural synchronization generating enhanced ERP source activity.

As for primary visuospatial consciousness, high-resolution EEG/MEG recordings from primary visual cortex are needed for a better understanding of the early stages of the processing of face stimuli. Sandberg et al. (2013) used MEG recordings during a binocular rivalry task with competing faces and gratings. Primary visual consciousness of faces was associated with enhanced occipital event-related magnetic fields (ERMFs) peaking at about 180 ms (M170) and 260 ms post-stimulus (P2 m).

Summing up, our “face expression” experiment showed that the primary visual consciousness of emoticons was associated with a negative ERP component named N170. Source analysis suggested an enhanced cortical neural synchronization in a distributed frontal and parietal network, not including temporal face processing cortex (Babiloni et al., 2010). These findings complement previous evidence showing that passive conscious perception of uncued visual stimuli was related to the so-called “visual awareness negativity” (VAN) over occipito-temporal electrodes from 200–400 ms post-stimulus (Koivisto et al., 2005; Koivisto and Revonsuo, 2008). More research with different semantic, emotional, and pictorial stimulus features is required to understand the neurophysiological meaning of the spatial localization of the effects in the present and mentioned studies.

## The Experiment on the ERPs Accompanying the Primary Consciousness of Written Words

**Figure 4:**
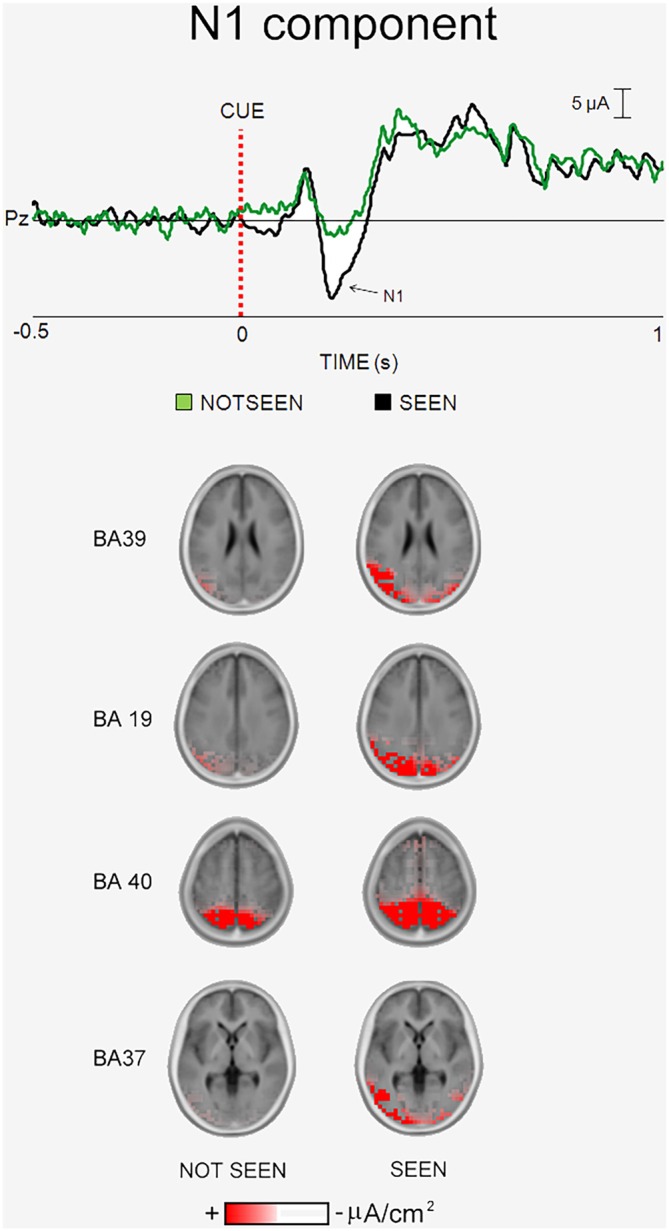
**Core results of the experimental paradigm used in the study of Babiloni et al. (under major revision in 2016).** Top: waveforms of the grand averaged (*N* = 17) ERPs are shown at Pz electrode for the “not seen” and “seen” trials. Bottom: the grand averaged eLORETA solutions modeling the distributed source activity for N1 component of the ERPs are shown. These solutions were based on the EEG voltages recorded from all 56 recording electrodes at the N1 peak latency. They concerned the “not seen” and “seen” trials averaged separately.

Figure 4 illustrates the main results of our “written word” experiment on the ERPs accompanying primary visual consciousness (Babiloni et al., under major revision in 2016). As mentioned above, the cue stimulus feature of interest was the reading of four common Italian words, two denoting living beings (“cane”, “mulo”; English: “dog” and “mule”) and two denoting not living beings (“pane”, “muro”; English: “bread” and “wall”). The words of living and not living beings differed only in one single letter. The post-stimulus ERP waveforms showed the highest amplitude at the parietal and temporal electrodes. In Figure 4, the ERP waveforms refer to Pz electrode (10–20 system) and unveil four main components, namely the P1, “N1”, P2 and P3. The peaks of these components were well visible in the ERPs formed averaging separately the “seen” and “not seen” trials. As for the other two experiments, there was the lack of statistical difference (*p* > 0.05) in the latency of the P1, “N1”, P2 and P3 peaks when the ERPs accompanying the “seen” trials were compared with those accompanying the “not seen” trials.

This result confirmed those of the “visuospatial” and “face expression” experiments and points to the same timing of cortical neural synchronization in the “seen” and “not seen” trials, regardless the primary visual consciousness. Again, the reaction time of the “go stimuli” was faster in the “seen” than the “not seen” trials, confirming the general concept that the primary consciousness was associated with an enhanced information processing of visual stimuli.

When did the cortical neural synchronization show some difference in relation to the primary consciousness of written words? In the “written words” experiment, the parietal “N1” component peaked about at 230 ms post-stimulus. This ERP component was the one that showed the highest difference between the “seen” and “not seen” trials (a higher amplitude in former than the latter, *p* < 0.05). This result suggests that human brain does process written words with enhanced cortical neural synchronization around 230 ms post-stimulus, possibly in association with the primary consciousness. As for the N170 of the face expressions (180–200 ms post-stimulus), the “N1” peak latency was earlier than that of the P3 peak in “visuospatial” experiment (e.g., 400 ms post-stimulus). This difference is suggestive of fast information processing for the primary consciousness of the written words, maybe for their intense learning along the whole life span (Batty and Taylor, 2003).

Where did the cortical neural synchronization show some differences in relation to the primary consciousness of written words? As the main finding, the source analysis of the “N1” component pointed to higher (eLORETA) current density in left parietal, occipital, and temporal areas in the primary consciousness of the written words (*p* < 0.05, Figure 4). This result suggests that human brain does process written words with an early (i.e., 230 ms post-stimulus) enhanced cortical neural synchronization in a left dorsal and ventral streams formed by occipital, parietal, and temporal areas. Namely, a cortical network different from those involved in the primary consciousness of visuospatial and schematic face expressions.

The results of the “written words” experiment extend to the cortical source space previous evidence showing early ERP components induced by the visual presentation of words within an “attentional blink” paradigm (Vogel et al., 1998; Sergent et al., 2005). In the “attentional blink”, two visual stimuli are presented in quick sequence, with the lack of conscious perception of the second one due to the residual cortical processing of the first stimulus. In our experiment, the effect of the primary visual consciousness was observed on “N1” component peaking at 230 ms post-stimulus. We put between brackets N1 as its latency is quite late for the typical N1 of visual evoked potentials. Rather, it shows the features of the so-called “visual awareness negativity” of the ERPs accompanying primary consciousness (see Railo et al., 2011 for a review).

Previous studies have shown that early visual P1 and N1 components, which were evoked by a probe flash (first stimulus) concomitant to the presentation of the letter (second stimulus), were preserved during the period when the stimulus report was affected by the “attentional blink” (Vogel et al., 1998). Furthermore, it has been shown that intact early scalp potentials (P1 and N1) were evoked by “not seen” words (second stimulus), suggesting that these ERP components were not the primary correlates of conscious perception in the “attentional blink” of words (Sergent et al., 2005). In line with the results of our “written words” experiment, a negativity peaking in the scalp parietal regions at about 270 ms post-stimulus was higher in amplitude in the “seen” than “not seen” words of the “attentional blink” paradigm (Sergent et al., 2005).

More recently, Melloni et al. (2011) using a letter recognition task reported that stimulus expectancy can reduce the latency of neuronal correlates of primary visual consciousness. Those authors used a paradigm manipulating the physical level of stimulus visibility and the stimulus expectancy parametrically. The ERP difference between “seen” and “not seen” trials were observed at the latency range of P3 component when there was a negligible stimulus expectancy (Melloni et al., 2011). In contrast, the ERP difference between “seen” and “not seen” trials was observed already at the P2 latency range when stimulus expectation was high. In that study (Melloni et al., 2011), the P2 amplitude decreased as the awareness of the stimulus increased (i.e., the amplitude of P2 was higher for the “not seen” trials than the “seen” trials). This result is apparently at odds with the literature regarding consciousness and requires future studies to be elucidated. We can provide just a tentative explanation in the following. In general, P2 component reflects the comparison of sensory inputs and stored memory (Luck and Hillyard, 1994). Therefore, it can be speculated that the high amplitude of P2 for the “not seen” trials (Melloni et al., 2011) was probably due to a mismatch between the high expectancy of the stimulus appearance generated by the experimental procedure (decreasing stimulus visibility in a sequence) and the missed stimulus detection (i.e., “not seen” trials). This effect on the P2 component was not observed in our “written words” experiment (Babiloni et al., 2014), reasonably due to the negligible effect of learning, stimulus expectancy, and cognitive load. Indeed, the duration of the cue stimuli in our experiment was determined subject-by-subject with a preliminary control test (no EEG recording), designed to minimize the effects of learning. Afterward, the physical stimulus features remained fixed during the whole EEG experimental session. Finally, results of a control experiment exhibited the lack of statistical differences among the mean percentages of “seen” trials in three different periods of the EEG experimental session, thus confirming the absence of a learning or fatigue effect.

## Conclusions and Future Directions

This article discussed the results of three ERP experiments of our group collectively testing the hypothesis that primary visual consciousness (i.e., subjective self-report of the stimulus) is related to an enhanced cortical neural synchronization as a function of stimulus features. The experimental design compared the ERP peak latency and sources between the “seen trials” and “not seen trials”, respectively related and unrelated to the primary visual consciousness. Three salient features of visual stimuli were considered (visuospatial, emotional face expression, and written words).

Results showed the typical ERP components following simple visual stimuli in both “seen” and the “not seen” trials. In all three experiments, there was no statistical difference (*p* > 0.05) in the ERP peak latencies between the “seen” and “not seen” trials. This finding suggests a similar timing of the cortical neural synchronization in the “seen” and “not seen” trials, regardless the primary visual consciousness.

In contrast, the ERP source analysis showed differences between the “seen” and “not seen” trials. For the visuospatial stimuli, the primary consciousness was related to higher activity in dorsal occipital and parietal sources at about 400 ms post-stimulus. For the emotional face expressions, there was greater activity in parietal and frontal sources at about 180 ms post-stimulus. For the written letters, there was higher activity in left occipital, parietal, and temporal sources at about 230 ms post-stimulus. These hint that primary visual consciousness is associated with an enhanced cortical neural synchronization having entirely different spatiotemporal characteristics as a function of the features of the visual stimuli and possibly, the corresponding qualia (i.e., visuospatial, face expression, and words). In this framework, the dorsal visual stream may be synchronized in association with the primary consciousness of visuospatial and emotional face contents, while both the dorsal and ventral visual streams may be synchronized in association with the primary consciousness of linguistic contents (Milner and Goodale, 2006).

The results of the three experiments suggest the inclusion of the neurophysiological mechanism of the cortical neural synchronization in the theories of consciousness proposed by the “localizationists” quoted in the Introduction of this article. The magnitude of the cortical neural synchronization within a specialized network may lead to the vividness of the primary consciousness of a given stimulus feature and relative “quale”. In this line of reasoning, the ensemble of the cortical neural networks underpinning the single visual features would constitute a sort of multi-dimensional palette of colors, shapes, regions of the visual field, movements, emotional face expressions, and words. The synchronization of one or more of these cortical neural networks, each with its peculiar timing, would produce the primary consciousness of one or more of the visual features of the scene.

Nevertheless, some *limitations and methodological remarks* of our studies should be considered. Firstly, primary consciousness should be not considered as an instantaneous mental experience to be related with one peak of a local neural concomitant. Rather, it should be considered as a progressive build-up phenomenon, since it reasonably emerges from relatively long integrative neural processes (i.e., hundreds of milliseconds), which might be related to fluctuating information flux and temporal synchronization among different brain regions (Tononi, 2001, 2004; Wyart et al., 2012).

Secondly, although different procedures to control for the effects of learning and attention were adopted in the present experiments, it is possible that some strange variables could affect the reliability of our data. In order to overcome this problem a better procedure might be the use of more fine-grained scales to rate the visibility of the stimuli, including different levels of confidence about the visibility reports, such as the four point PAS (see Melloni et al., 2011).

Thirdly, EEG source analyses in our experiments were carried out with LORETA/eLORETA whose spatial resolution is much lower (centimeters) than that of positron emission tomography (PET) and fMRI (millimeters). Of note, a better solution for source localization can be achieved by eLORETA and sLORETA than original LORETA (Pascual-Marqui et al., 2002; Pascual-Marqui, 2007). Indeed, both sLORETA and eLORETA showed low spatial resolution but zero localization error in the presence of measurement and biological noise in simulation studies (Pascual-Marqui et al., 2002; Pascual-Marqui, 2007). Furthermore, it was reported a better source location by eLORETA than sLORETA (Canuet et al., 2011).

Finally, although our analyses were limited to just one neural concomitant of the consciousness phenomenon (ERP sources), other interesting developments of our general view have been carried out, such as the influence of top-down processes on the primary visual consciousness in the visuospatial experiment (see above) or regard the causal role of the cortical neural synchronization. In this vein, an experiment of our group showed that an inhibitory low-frequency (1 Hz) repetitive transcranial magnetic stimulation (rTMS) over right or left posterior parietal areas significantly reduced the percentage of visual stimuli consciously perceived, thus confirming the causal contribution of those areas in the primary visuospatial consciousness (Babiloni et al., 2007). A new story of the consciousness has just started.

## Funding

The present article developed a general neurophysiological interpretation of the results of three original studies (cited in the main body text) financially supported by the Department of Physiology and Pharmacology of the University of Rome “La Sapienza” and the Association Fatebenefratelli for Research (AFaR). We thank all Authors of those original studies, with a special mention to Dr. Fabrizio Vecchio and Prof. Paolo M. Rossini for their precious contribution.

## Author Contributions

All the authors developed the literature review. CB, AS, and AB gave their contributions to the conception and design of the review. CB and AB contributed to the writing of the first draft of the manuscript. CB, AS, SC, JCM-C and CDP contributed in drafting the work and revising it critically for important intellectual content. SC, CDP and AB built the figures and the reference list. All the authors collaborated in the revision of the final manuscript.

## Conflict of Interest Statement

The authors declare that the research was conducted in the absence of any commercial or financial relationships that could be construed as a potential conflict of interest.

## Footnotes

^1^http://www.uzh.ch/keyinst/loreta.htm
